# The value of chest CT in the differential diagnosis of benign and malignant pulmonary nodules: a meta-analysis

**DOI:** 10.3389/fmed.2026.1854231

**Published:** 2026-05-28

**Authors:** Risheng Chen, MingJuan Fu, Shun Hu, Yuanyuan Zhong

**Affiliations:** 1 School of Medical Imaging, Changsha Medical University, Changsha, Hunan, China; 2 Department of Pharmacy, The Third People’s Hospital of Yunnan Province, Kunming, Yunnan, China

**Keywords:** chest CT, diagnosis, lung cancer, meta-analysis, multiple pulmonary nodules

## Abstract

**Background:**

This study was conducted to systematically evaluate the diagnostic value of computed tomography (CT) in differentiating benign and malignant pulmonary nodules through a meta-analysis, thereby providing a solid evidence-based foundation for clinical diagnosis and treatment decisions.

**Methods:**

We searched the PubMed, Web of Science, Embase, Medline, China National Knowledge Infrastructure (CNKI), and Cochrane Library databases for relevant studies published up to December 2025 and performed a meta-analysis using Review Manager v.5.4.

**Results:**

A total of 28 retrospective analyses and 19 prospective studies were included, comprising 4,705 patients with pulmonary nodules. The pooled sensitivity of CT was 0.90 (95% confidence interval [CI]: 0.89–0.91; *p* < 0.001), while the pooled specificity was 0.74 (95% CI: 0.72–0.76; *p* < 0.001). The positive likelihood ratio was 3.28 (95% CI: 2.81–3.83; *p* < 0.001), the negative likelihood ratio was 0.13 (95% CI: 0.10–0.17; *p* < 0.001), and the diagnostic odds ratio (DOR) was 33.20 (95% CI: 23.52–46.87; *p* < 0.001). The area under the summary receiver operating characteristic (SROC) curve was 0.9177.

**Conclusion:**

These findings indicate that CT has a high diagnostic value in differentiating benign from malignant pulmonary nodules and is an important method for evaluating pulmonary nodules in clinical practice.

**Systematic review registration:**

https://www.crd.york.ac.uk/PROSPERO/view/CRD420261277036, Identifier: CRD420261277036.

## Highlights

- Chest CT showed high accuracy for differentiating benign and malignant pulmonary nodules (pooled sensitivity: 90%, specificity: 74%, and AUC: 0.9177).
- This meta-analysis provided robust, stratified evidence on enhanced vs. non-enhanced CT performance.
- CT should be the first-line tool for nodule evaluation, with protocol selection tailored to patient risk to help reduce unnecessary procedures.

## 1 Introduction

A pulmonary nodule is defined as an abnormal lesion in the lung that is less than 3 cm in diameter (1). Pulmonary nodules can be categorized into two subtypes: benign and malignant. The majority of benign pulmonary nodules present with well-defined margins, whereas malignant pulmonary nodules are commonly characterized by spiculation and lobulation (2) and are associated with a high risk of developing into lung cancer. Lung cancer has been recognized as the most frequently diagnosed cancer (12.4% of global cancers), and it remains the leading cause of cancer-related mortality worldwide, accounting for approximately 1.8 million deaths per year, according to the Global Cancer Observatory (GLOBOCAN) 2022 estimates (3). Therefore, early detection and accurate differentiation of benign and malignant pulmonary nodules are critical for improving the prognosis of patients. Although the diagnostic value of CT for pulmonary nodules has been established, previous meta-analyses have had specific limitations, including small sample sizes and a lack of subgroup analyses. The present study addresses these gaps by expanding the sample size and conducting subgroup analyses.

Currently, the pathological examination of percutaneous biopsy and postoperative specimens serves as the gold standard for distinguishing benign from malignant pulmonary nodules. However, these procedures are invasive and have inherent risks, including pneumothorax, hemorrhage, and infection. In contrast, imaging modalities, such as digital radiography (DR), computed tomography (CT), and magnetic resonance imaging (MRI), are non-invasive. Among these techniques, DR plays a limited role in the assessment of pulmonary nodules due to its low-contrast resolution, insufficient soft tissue visualization capability, and two-dimensional imaging nature. MRI is constrained by low proton density, numerous air–tissue interfaces, and respiratory or cardiac motion artifacts due to its prolonged scanning duration (4). A previous meta-analysis indicated that MRI is inferior to CT in the detection of pulmonary nodules (5). For MRI and CT, the pooled sensitivity was 0.91 (95% confidence interval [CI], 0.80–0.96) and 1.00 (95% CI, 0.95–1.00), and the pooled specificity (95% CI) was 0.76 (95% CI, 0.58–0.87) and 0.99 (95% CI, 0.78–1.00), respectively. Therefore, chest CT has emerged as the preferred imaging modality for the evaluation of pulmonary nodules because of its high spatial resolution, excellent soft tissue contrast, and fast temporal resolution (6).

Although the diagnostic value of CT for pulmonary nodules has been established, previous meta-analyses have had specific limitations, including small sample sizes and a lack of subgroup analyses by enhancement. Meanwhile, robust evidence characterizing the diagnostic value of CT in differentiating benign from malignant pulmonary nodules remains scarce. The present study addresses these gaps by expanding the sample size and conducting subgroup analyses. Specifically, this comprehensive diagnostic meta-analysis systematically synthesizes relevant clinical studies to quantitatively assess the diagnostic performance of CT, with the aim of providing clinicians with evidence-based recommendations to optimize therapeutic decision-making and improve clinical outcomes in patients with pulmonary nodules.

## 2 Materials and methods

The study design and implementation of this meta-analysis were conducted in full compliance with the Preferred Reporting Items for Systematic Reviews and Meta-Analyses (PRISMA) statement. This review was registered in the International Prospective Register of Systematic Reviews (PROSPERO) database (CRD420261277036).

### 2.1 Search strategy

All relevant studies in the PubMed, Embase, CNKI, MEDLINE, Web of Science, and Cochrane Library databases published up to December 2025 were retrieved. This research adopted a retrieval strategy combining subject headings with free-text terms, including “Computed Tomography,” “Dynamic contrast-enhanced computed tomography”, “CT”, “DCE-CT”, “Solitary lung nodule”, “Pulmonary nodules”, “Malignant”, and “Diagnosis.” After independent literature screening by two reviewers based on the predefined inclusion and exclusion criteria, the quality assessment for the meta-analysis was performed using Review Manager v5.4.

### 2.2 Inclusion and exclusion criteria

To ensure accuracy and reliability, studies were selected for the meta-analysis using the following inclusion and exclusion criteria. The inclusion criteria were as follows: (a) clinical trials evaluating the use of CT for the diagnosis of pulmonary nodules; (b) studies differentiating between benign and malignant pulmonary nodules; (c) studies with extractable diagnostic indicators [e.g., the number of true positives (TPs), true negatives (TNs), false positives (FPs), and false negatives (FNs)]; (d) studies published in English; and (e) studies evaluating pulmonary nodules at the time of initial detection on chest CT. The exclusion criteria were as follows: (a) irrelevant articles; (b) animal studies; (c) non-comparative studies; (d) reviews; and (e) studies that did not report sufficient data for statistical analysis.

### 2.3 Data extraction

Two researchers independently extracted the basic information of eligible articles, including authors, year of publication, region, scanning method, study design, and patient sample size, as well as imaging outcome data (true-positive, false-positive, true-negative, and false-negative results). Any discrepancies were resolved through consensus review by a third investigator.

### 2.4 Timing of nodule evaluation

In all included studies, the characterization of solitary pulmonary nodules was performed at the initial CT detection rather than during subsequent imaging follow-up.

### 2.5 Quality assessment

**Figure 1:**
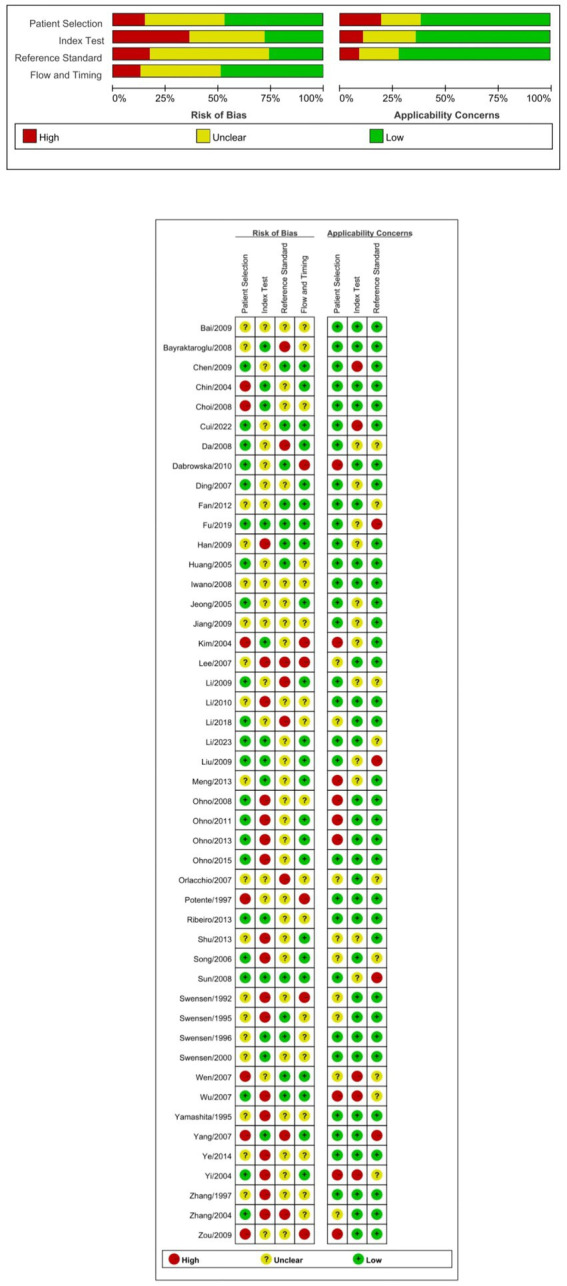
Quality assessment of the included studies using the QUADAS-2 tool.

In this step, the methodological quality of the included studies was assessed using the Quality Assessment of Diagnostic Accuracy Studies-2 (QUADAS-2) tool (7), which comprises four key domains: patient selection, index test, reference standard, and flow and timing (Figure 1). All domains were evaluated with respect to the risk of bias. This assessment was aimed at enhancing the rigor of evaluating diagnostic studies. Each domain was systematically rated as “low risk of bias”, “high risk of bias”, or “unclear risk of bias” based on predefined, study-specific criteria aligned with the tool’s guidelines. All included studies were assessed by three reviewers independently.

### 2.6 Statistical analyses

Review Manager v.5.4 and MetaDiSc v1.4 were used for all statistical analyses. In this meta-analysis, relevant data were extracted from all included studies, and 2 × 2 contingency tables were constructed, including the values of true positive (TP), true negative (TN), false positive (FP), and false negative (FN). Pooled estimates of sensitivity, specificity, and diagnostic odds ratio (DOR) with their corresponding 95% confidence intervals (CIs) were calculated to derive comprehensive results. Sensitivity was defined as the proportion of patients with the disease who were correctly identified as positive, with the formula: TP/(TP + FN). Specificity was defined as the proportion of disease-free subjects who were correctly identified as negative, calculated as TN/(TN + FP). DOR was an indicator reflecting diagnostic performance; a DOR value greater than 1 indicated better discriminative ability of the diagnostic test, and its formula was (TP × TN)/(FP × FN). Summary receiver operating characteristic (SROC) curves were plotted to illustrate the overall performance of the index test. The closer the curve was to the upper left corner of the SROC space, the higher the overall performance. Heterogeneity in sensitivity and specificity across studies was assessed using the chi-square-based Cochrane *Q* test and *I*^2^ inconsistency test. Significant heterogeneity was considered present if *I*^2^ was > 50% or *p*-value of <0.10; in such cases, a random-effects model was applied, whereas a fixed-effects model was used otherwise.

## 3 Results

### 3.1 Study characteristics

**Figure 2:**
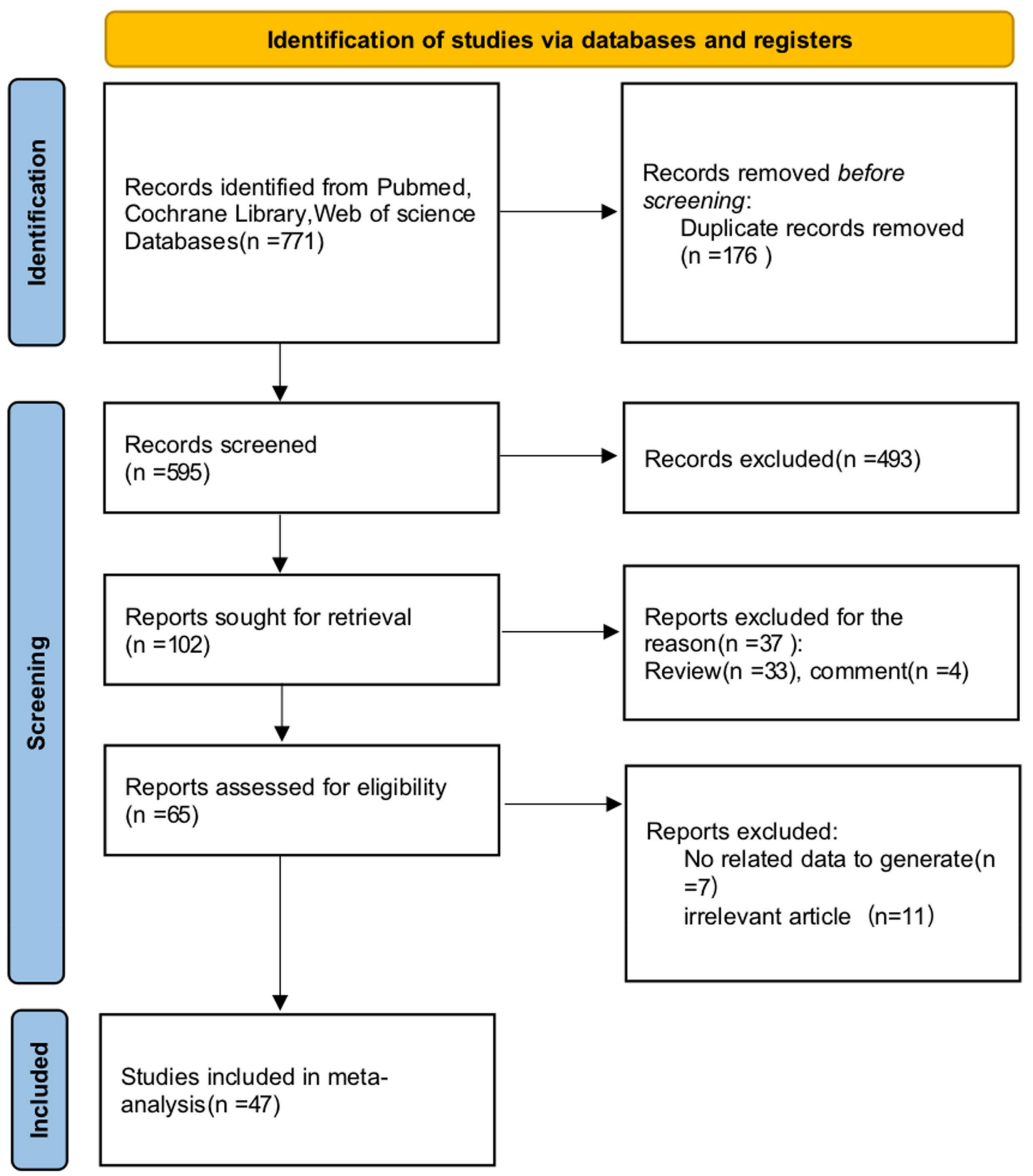
The flowchart of this study.

The initial search strategy identified 771 potentially relevant studies, of which 47 analyses (8–53) were ultimately included in the final meta-analysis (Figure 2 and Table 1). This study included 4,705 patients with pulmonary nodules.

**Table 1:** Main characteristics of the included studies in the meta-analysis.

Author/year	Scanning method	District	Slice thickness(mm)	CT protocols	Design	TP	FP	FN	TN	Patients	F/M
Swensen/1992 (8)	Enhanced	America	1.5–2.0	SDCT	Retrospective	23	1	0	6	30	7/23
Yamashita/1995 (9)	Enhanced	Japan	2.0	SDCT	Retrospective	18	1	0	13	32	11/21
Swensen/1995 (10)	Enhanced	America	1.5–3.0	SDCT	Retrospective	111	12	0	40	163	69/94
Swensen/1996 (11)	Enhanced	America	1.0–2.0	SDCT	Retrospective	51	14	1	41	107	50/57
Swensen/2000 (12)	Enhanced	America	2.0	SDCT	Prospective	167	78	4	107	356	181/175
Potente/1997 (13)	Enhanced	Italy	1.0	SDCT	Retrospective	17	2	0	6	25	5/20
Zhang/1997 (14)	Enhanced	China	5.0	SDCT	Retrospective	40	7	2	16	65	25/40
Kim/2004 (16)	Enhanced	Korea	3.0	SDCT	Prospective	17	10	2	21	50	18/32
Jeong/2005 (17)	Enhanced	Korea	2.5	MDCT	Retrospective	46	6	3	52	107	45/62
Orlacchio/2007 (18)	Enhanced	Italy	1.25	MDCT	Prospective	24	0	2	30	56	20/36
Lee/2007 (19)	Enhanced	Korea	2.5	MDCT	Prospective	212	52	25	197	486	187/299
Ohno/2008 (20)	Enhanced	Japan	2.5	MDCT	Prospective	142	21	10	29	175	83/92
Choi/2008 (21)	Enhanced	Korea	2.0	SDCT	Retrospective	12	3	1	24	40	11/29
Bayraktaroglu/2008 (22)	Enhanced	Turkey	2.0	SDCT	Retrospective	9	2	0	11	22	10/12
Bai/2009 (23)	Non-enhanced	China	3.0	MDCT	Retrospective	34	16	2	16	68	30/38
Jiang/2009 (24)	Enhanced	China	2.5	MDCT	Retrospective	26	5	2	18	51	20/31
Dabrowska/2010 (25)	Enhanced	Poland	3.0	SDCT	Retrospective	23	9	0	8	40	13/27
Li/2010 (26)	Non-enhanced	China	3.0	MDCT	Prospective	43	2	3	20	77	25/52
Ohno/2011 (27)	Non-enhanced	Japan	2.0	MDCT	Prospective	42	7	1	26	50	32/45
Ohno/2013 (28)	Enhanced	Japan	2.0	MDCT	Prospective	49	8	8	31	52	37/47
Ohno/2015 (29)	Enhanced	Japan	2.0	MDCT	Prospective	123	25	10	60	198	87/111
Shu/2013 (30)	Non-enhanced	China	1.25	MDCT	Prospective	68	12	8	56	144	X/X
Ribeiro/2013 (31)	Enhanced	Brazil	3.0	SDCT	Retrospective	4	8	1	10	23	10/13
Li/2009 (32)	Enhanced	China	2.0	MDCT	Retrospective	34	0	11	8	63	24/45
Fan/2012 (33)	Enhanced	China	1.25	MDCT	Retrospective	57	4	7	14	82	47/35
Chen/2009 (34)	Enhanced	China	1.25	MDCT	Retrospective	109	35	26	100	352	111/241
da Silva/2008 (35)	Non-enhanced	Brazil	1.0	MDCT	Prospective	7	0	3	29	39	X/X
Ding/2007 (36)	Non-enhanced	China	1.25	MDCT	Retrospective	21	2	0	10	32	17/22
Han/2009 (37)	Enhanced	China	5.0	SDCT	Retrospective	20	8	1	10	39	17/22
Huang/2005 (38)	Enhanced	China	3.0	SDCT	Retrospective	38	6	0	7	51	10/41
Iwano/2008 (39)	Non-enhanced	Japan	1.0	MDCT	Retrospective	40	12	12	43	107	48/58
Liu/2009 (40)	Non-enhanced	China	X	X	Retrospective	44	18	16	42	120	97/117
Song/2006 (41)	Enhanced	China	10	SDCT	Retrospective	69	12	12	18	111	28/83
Sun/2008 (42)	Enhanced	China	5.0	SDCT	Prospective	28	4	8	21	61	26/35
Wen/2007 (43)	Enhanced	China	X	SDCT	Retrospective	39	17	7	11	74	26/48
Ye/2014 (44)	Enhanced	China	1.25	MDCT	Prospective	31	7	21	28	87	28/59
Li/2018 (45)	Non-enhanced	China	X	MDCT	Prospective	44	0	2	19	65	X/X
Meng/2013 (46)	Non-enhanced	China	2.0	MDCT	Prospective	31	0	1	8	40	X/X
Fu/2019 (47)	Non-enhanced	China	2.0	MDCT	Prospective	86	0	10	36	136	47/89
Li/2023 (48)	Non-enhanced	China	2.0	MDCT	Prospective	62	0	8	30	100	38/62
Wu/2007 (49)	Enhanced	China	X	X	Retrospective	40	8	10	30	88	36/52
Yang/2007 (50)	Non-enhanced	China	X	X	Prospective	45	7	14	20	86	31/55
Yi/2004 (15)	Enhanced	Korea	1.25	MDCT	Retrospective	69	28	1	33	131	49/82
Zhang/2004 (51)	Enhanced	China	X	X	Retrospective	50	12	2	16	80	30/50
Zou/2009 (52)	Enhanced	China	2.0	MDCT	Retrospective	47	9	11	38	105	46/59
Cui 2022 (53)	Non-enhanced	China	1.25	MDCT	Retrospective	89	1	4	14	108	62/46

### 3.2 Meta-analysis results

**Figure 3:**
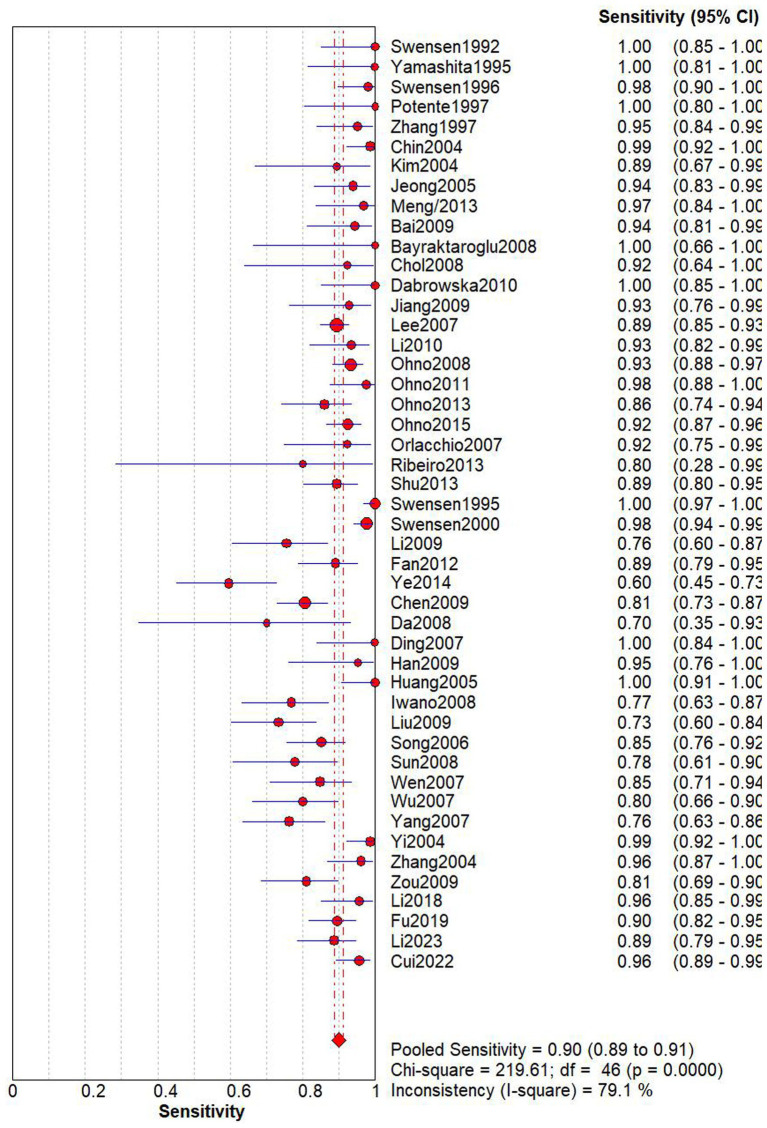
Forest plot of estimates of sensitivity.

**Figure 4:**
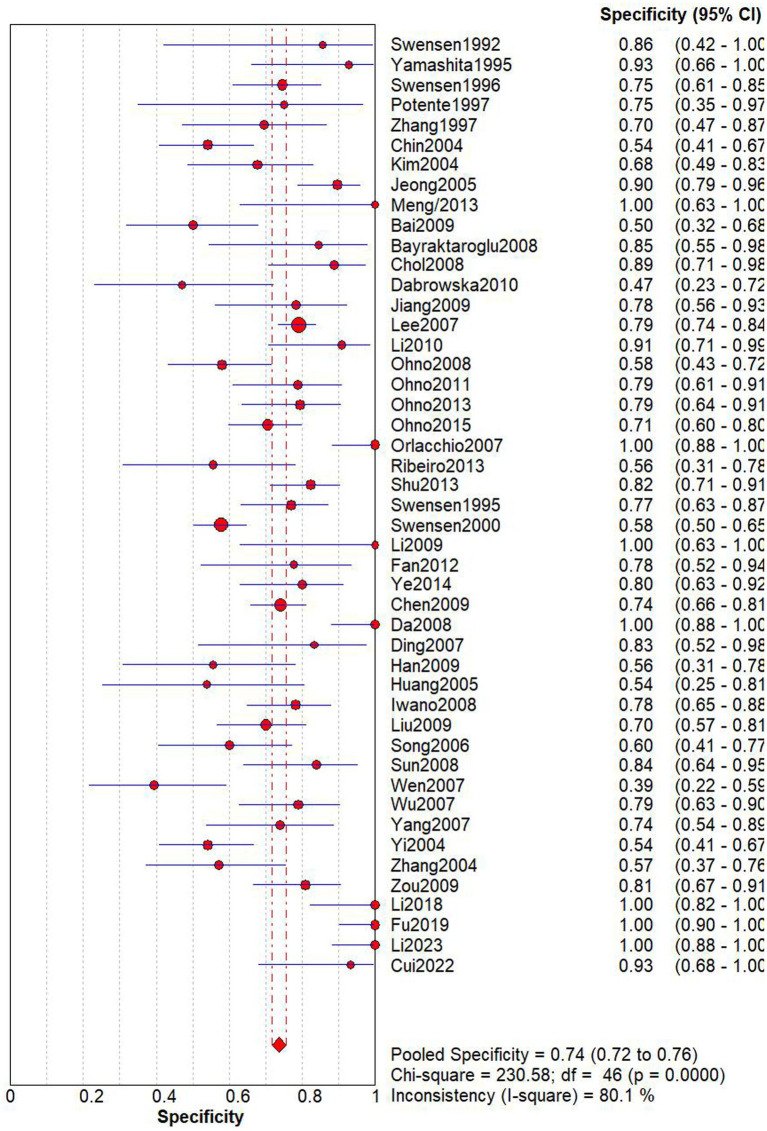
Forest plot of estimates of specificity.

Figures 3, 4 show the forest plot of sensitivity and specificity for CT scanning in the diagnosis of benign and malignant pulmonary nodules. The sensitivity was 0.90 (95% confidence interval [CI]: 0.89 to 0.91; *p* < 0.001), and the specificity was 0.74 (95% CI: 0.72–0.76; *p* < 0.001). The results also noted that the positive likelihood ratio (LR) was 3.28 (95% CI: 2.81–3.83; *p* < 0.001), the negative LR was 0.13 (95%CI: 0.10–0.17; *p* < 0.001), and the DOR was 33.20 (95%CI: 23.52–46.87; *p* < 0.001).

**Figure 5:**
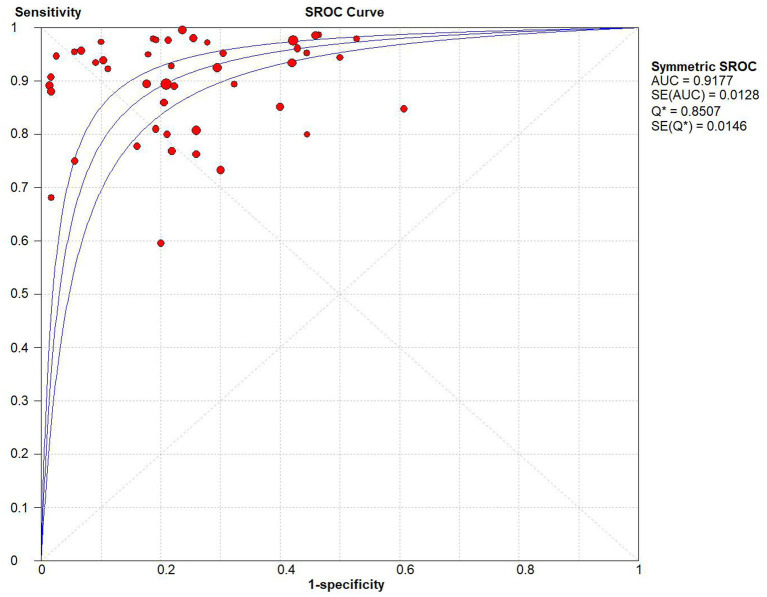
SROC curve for CT scanning in the diagnosis of pulmonary nodules.

The Spearman correlation coefficient indicated that no significant correlation was observed between sensitivity and specificity (*r* = 0.247, *p* = 0.09), suggesting the absence of a threshold effect. The SROC curve offered a comprehensive summary of diagnostic test performance and reflected the balance between sensitivity and specificity. A symmetrical SROC curve was selected for the analysis, as the statistical test for the b-coefficient indicated no significant asymmetry (*p* = 0.66). A plot of the SROC curve for CT scanning in the diagnosis of pulmonary nodules, depicting true-positive vs. false-positive rates from individual studies is presented in Figure 5.

### 3.3 Subgroup analyses

Subgroup analyses were performed according to the scanning method (enhanced vs. non-enhanced), geographic region (Asia vs. Western countries), patient’s sample size (<100 patients vs. ≥100 patients), study design (retrospective vs. prospective), slice thickness (≤2 mm vs. > 2 mm), and CT protocol [single-detector vs. multidetector CT (MDCT)] features in models (Table 2). Only studies with complete true-positive, false-positive, true-negative, and false-negative imaging data were included in the subgroup analyses to guarantee comparability of diagnostic accuracy estimates across subgroups. Subgroup analyses indicated that enhanced CT exhibited lower specificity than non-enhanced CT (71% vs. 83%), whereas enhanced CT exhibited a higher sensitivity than non-enhanced CT (91% vs. 89%). The small sample group exhibited a higher specificity than the large sample group (78% vs. 73%). Regarding the slice thickness, studies using a slice thickness of ≤2 mm exhibited significantly higher specificity (75% vs. 72%) than those with a slice thickness of >2 mm. Conversely, the subgroup using a slice thickness of >2 mm exhibited higher sensitivity (93% vs. 89%).

**Table 2:** Subgroup analyses.

Subgroup	Sensitivity	Specificity	Positive LR	Negative LR	DOR
Enhanced	0.91(0.89–0.92)	0.71(0.69–0.73)	2.98(2.55–3.48)	0.13(0.10–0.18)	27.82(19.31–40.08)
Non-enhanced	0.89(0.86–0.91)	0.83(0.79–0.86)	5.35(3.30–8.68)	0.13(0.09–0.20)	60.38(25.99–140.26)
Asia	0.89(0.87–0.90)	0.75(0.73–0.77)	3.32(2.78–3.96)	0.15(0.12–0.19)	27.91(19.58–39.77)
Western countries	0.98(0.96–0.99)	0.70(0.65–0.74)	3.24(2.23–4.71)	0.07(0.03–0.17)	82.33(36.57–185.35)
Sample size (<100)	0.89(0.87–0.91)	0.78(0.72–0.79)	3.41(2.65–4.39)	0.14(0.10–0.20)	32.68(20.00–53.40)
Sample size (≥100)	0.91(0.89–0.92)	0.73(0.70–0.75)	3.28(2.67–4.02)	0.12(0.09–0.17)	34.73(21.02–57.39)
Retrospective	0.90(0.88–0.92)	0.72(0.69–0.75)	3.02(2.50–3.65)	0.14(0.10–0.19)	29.21(18.22–46.82)
Prospective	0.90(0.86–0.92)	0.75(0.73–0.78)	3.82(2.90–5.03)	0.13(0.09–0.19)	38.69(23.67–63.27)
Slice thickness (≤2)	0.89(0.87–0.90)	0.75(0.73–0.77)	3.70(2.98–4.60)	0.14(0.11–0.19)	35.80(22.49–56.99)
Slice thickness (>2)	0.93(0.91–0.94)	0.72(0.68–0.75)	2.85(2.26–3.59)	0.12(0.80–0.17)	31.21(19.24–50.64)
Single-detector CT	0.91(0.90–0.93)	0.67(0.63–0.70)	2.69(2.25–3.21)	0.13(0.08–0.20)	25.35(14.30–44.94)
Multidetector CT	0.89(0.88–0.91)	0.78(0.75–0.80)	3.99(3.14–5.08)	0.13(0.10–0.18)	40.22(26.19–61.77)

### 3.4 Publication bias

**Figure 6:**
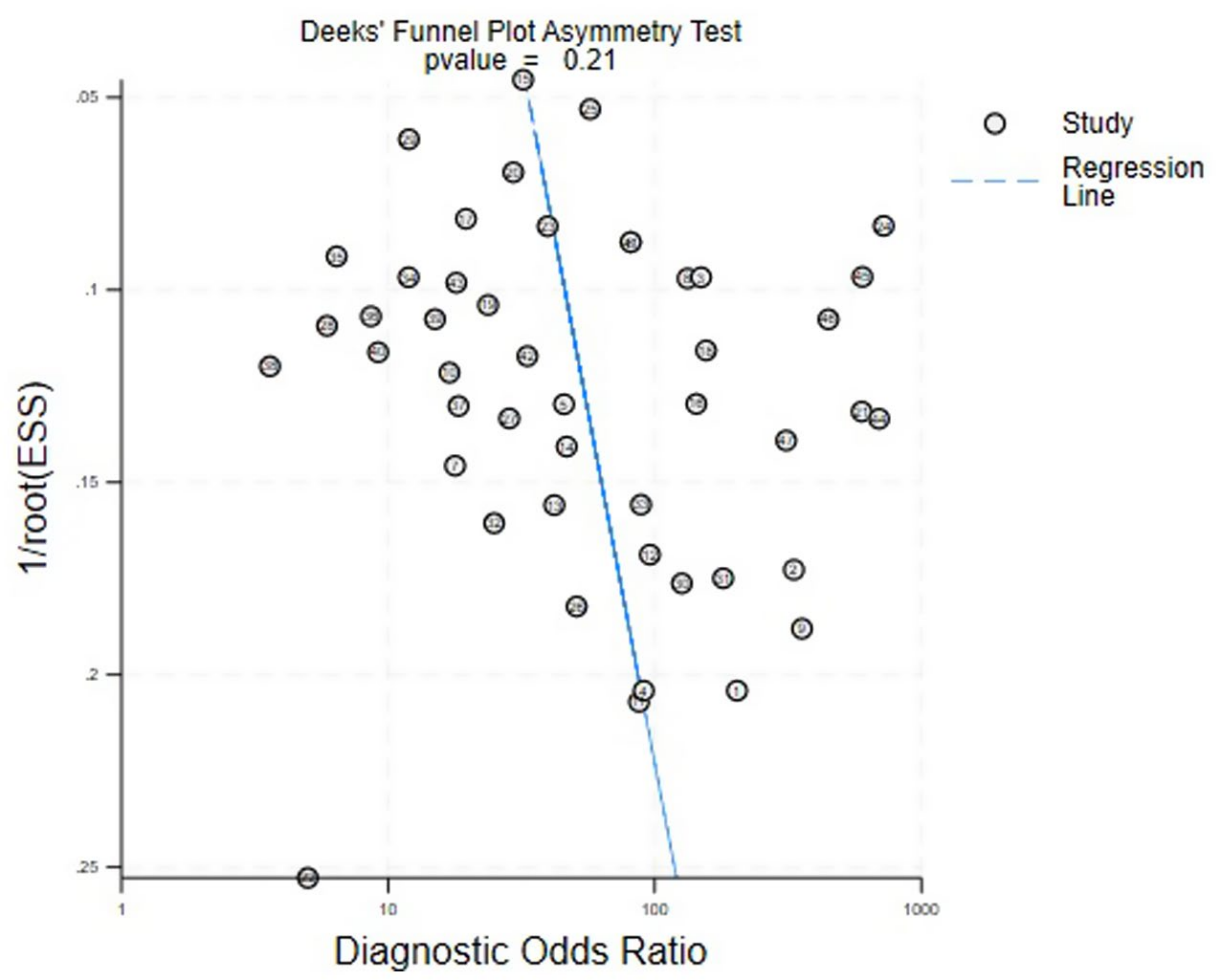
Funnel plot for publication bias.

Stata v18.0 was used for assessing publication bias, and the possibility of publication bias was evaluated utilizing Deeks’ funnel plot analysis, with the results presented in Figure 6. Significant publication bias was defined as *p*-value of <0.01, while no publication bias was identified when the *p*-value was >0.05. The funnel plot for publication bias showed *p* = 0.21; therefore, no evidence of publication bias was identified in the included studies.

## 4 Discussion

Accurate differentiation between benign and malignant pulmonary nodules was pivotal for the clinical management and prognostic stratification of affected patients. To overcome the shortcomings of previous relevant meta-analyses, such as limited sample size and lack of subgroup analysis, this study was designed to fill these research gaps. In this meta-analysis, we comprehensively evaluated the diagnostic performance of computed tomography (CT) for distinguishing benign from malignant pulmonary nodules and further quantified its true diagnostic efficiency to address the shortage of high-quality clinical evidence in this field. Pooled estimates yielded a sensitivity of 0.90 (95% confidence interval [CI]: 0.89–0.91), a specificity of 0.74 (95% CI: 0.72–0.76), a positive likelihood ratio (PLR) of 3.28 (95% CI: 2.81–3.83), a negative likelihood ratio (NLR) of 0.13 (95% CI: 0.10–0.17), and a diagnostic odds ratio (DOR) of 33.20 (95% CI: 23.52–46.87). These concrete indicator data confirmed that CT has a prominent screening capability for malignant pulmonary nodules and a reliable differential diagnostic capability. Collectively, these findings demonstrated that CT confers robust diagnostic value for the differentiation of benign and malignant pulmonary nodules. In clinical practice, these results could help clinicians formulate more precise diagnostic strategies; avoid excessive examination and unnecessary invasive biopsy for benign nodule patients to reduce the risk of complications such as pneumothorax, hemorrhage, and hemothorax; and achieve early screening and standardized treatment of malignant nodules, which could contribute to improving the overall clinical benefit for patients.

The above findings could be translated into clinical risk stratification and management pathways for pulmonary nodules. Given its high negative predictive value, CT effectively identifies low-risk patients who may be safely managed with routine surveillance. Intermediate-risk nodules warrant further investigation, including positron emission tomography (PET)/CT or short-interval CT follow-up in accordance with the Fleischner Society guidelines. For high-risk nodules with features highly suggestive of malignancy, our data support prompt proceeding to tissue sampling, such as CT-guided biopsy, bronchoscopy, or surgical resection. Furthermore, CT morphological features provide incremental diagnostic value beyond demographic and clinical variables in established risk prediction models, including the Mayo Clinic, Brock, and Herder models, reinforcing the central role of CT in integrated risk assessment. Notably, clinical applicability remains limited in specific scenarios, such as subsolid nodules with indeterminate characteristics and small nodules in high-risk patients, in which shared decision-making is imperative.

In comparison with the 2014 meta-analysis (54) assessing CT-based differentiation of benign and malignant pulmonary nodules, this study offers the distinct advantage through the implementation of more comprehensive subgroup analyses. These analyses encompassed geographic region (Asia vs. Western countries), scanning technique (enhanced vs. non-enhanced CT), and sample size stratification (<100 patients vs. ≥100 patients), which served to mitigate study heterogeneity to a certain extent and to improve the clinical applicability of the resultant findings. Compared with a prospective multi-center study published in 2021, Wu et al. (55) evaluated the diagnostic efficacy of CT in differentiating benign and malignant pulmonary nodules. Pooled sensitivity, specificity, and area under the SROC curve were 0.92 (95%CI, 0.89–0.95), 0.64 (95%CI, 0.54–0.74), and 0.91 (95%CI, 0.88–0.93), respectively. The pooled sensitivity, specificity, and area under the SROC curve derived from the present meta-analysis were closely concordant with the CT-related results of Wu et al.’s (55) study, thereby corroborating the robust diagnostic performance of CT for this clinical indication. From a technical standpoint, technological advancements, including thin-slice CT, high-resolution CT, and dual-energy CT, have refined lesion characterization and quantitative accuracy by virtue of thinner sectional imaging, improved spatial resolution, and multi-energy imaging capabilities. Despite these technical improvements, interstudy heterogeneity still existed, which was attributable to differences between regions, differences between enhanced and non-enhanced scans, and sample sizes.

Part of the heterogeneity observed in this study stemmed from variations in scanning protocols and study populations. A subgroup analysis demonstrated that non-enhanced CT yielded a significantly higher specificity than enhanced CT (0.83 vs. 0.71), alongside a concomitant higher positive likelihood ratio (5.35 vs. 2.98). This disparity may be attributable to contrast enhancement being a non-specific imaging finding, which could occur in benign inflammatory or tuberculous granulomas whose enhancement patterns overlap with those of malignant nodules, thereby resulting in an increased rate of false-positive diagnoses. In contrast, non-enhanced CT facilitates the clear identification of classic benign morphological features within nodules, such as popcorn calcification or fat density; these features enable the accurate exclusion of benign lesions, thereby reducing misdiagnosis and improving the true-negative rate. With respect to regional variability, diagnostic specificity was comparable between Asian and Western study populations (0.75 vs. 0.70), whereas sensitivity was marginally lower in Asian cohorts (0.89 vs. 0.98). This finding might be associated with the higher prevalence of pulmonary nodules in East Asian populations (3), in conjunction with potential regional differences in the diagnostic thresholds applied for pulmonary nodule characterization. An increased prevalence leads to a reduced sample size of non-cases, which is prone to underestimating specificity, while a lower diagnostic threshold elevates the number of false positives, thus lowering specificity. Regarding slice thickness, the subgroup with a slice thickness of ≤2 mm showed higher specificity (75% vs. 72%), while those with a slice thickness of >2 mm showed superior sensitivity (93% vs. 89%). This finding could possibly be explained by the fact that thin-section CT improves in-plane spatial resolution and permits clearer delineation of the marginal and internal morphological features of small pulmonary nodules, thereby facilitating more precise lesion characterization. Although thicker sections may mitigate partial volume averaging, they compromise detailed morphological evaluation, which contributes to the observed reduction in specificity. Regarding CT protocols, multidetector computed tomography (MDCT) showed significantly higher specificity than single-detector CT (SDCT) (78% vs. 67%). This could likely be explained by the advanced technical features of MDCT, including thinner collimation, faster image acquisition, and reliable multiplanar reconstruction (MPR), collectively improving the diagnostic performance of CT for differentiating benign from malignant pulmonary nodules. The CT features of malignant pulmonary nodules have a well-established pathological basis (56): lobulation reflects asymmetric tumor growth, spiculation is indicative of local parenchymal invasion, and specific calcification patterns correlate with distinct histological subtypes of lung cancer. In contrast, benign pulmonary lesions, including inflammatory granulomas, tuberculous foci, and fungal infections, frequently present as ground-glass or solid nodules with enhancement or morphological features that mimic malignant lesions, and this morphological overlap remains the primary cause of false-positive diagnoses in CT-based differentiation of pulmonary nodules.

The diagnostic findings of this study provided valuable imaging evidence to inform clinical risk stratification for distinguishing benign from malignant pulmonary nodules. They also further demonstrated that computed tomography (CT) examinations exhibit variable diagnostic performance according to scanning modality (enhanced vs. non-enhanced), geographic region, sample size, study design, slice thickness, and detector type, which could guide the rational selection of imaging approaches across distinct clinical scenarios.

Subgroup analyses in this study further stratified the diagnostic performance of CT across diverse clinical and demographic subgroups, offering clinically actionable risk stratification for patient management. Significantly, non-enhanced CT yielded superior specificity (0.83, 95% CI: 0.79–0.86) and diagnostic odds ratio (DOR = 60.38, 95% CI: 25.99–140.26) compared with enhanced CT, while both modalities maintained consistently high sensitivity (> 0.89). Furthermore, CT demonstrated exceptional sensitivity (0.98, 95% CI: 0.96–0.99) in Western populations, with stable diagnostic performance across strata of different sample sizes. Overall, these findings highlight the robust clinical utility of CT for risk stratification across heterogeneous patient cohorts. In resource-constrained healthcare settings, CT is a highly accessible and accurate diagnostic technique that facilitates optimized resource allocation, prioritization of high-risk individuals, and equitable provision of diagnostic care for underserved populations.

Chest CT enables high-resolution delineation of the intricate anatomical architecture and detailed morphological features of pulmonary nodules, representing the cornerstone imaging modality for the initial characterization and risk stratification of pulmonary nodules. By comparison, PET/CT yields complementary data regarding lesional glucose metabolic activity, thereby enabling a multidimensional, integrated diagnostic paradigm. For pulmonary nodules measuring >8 mm in diameter or those categorized as intermediate malignant risk, morphological assessment using chest CT confers particularly substantial clinical utility. In contrast, while PET/CT demonstrates superior overall specificity, its diagnostic sensitivity is substantially compromised for subcentimeter nodules and pure ground-glass nodules. The diagnostic performance of PET/CT is further constrained by a multitude of factors, including nodule dimension and histopathological subtype (e.g., adenocarcinoma *in situ* typically exhibits false-negative fluorodeoxyglucose uptake), as well as regional heterogeneity in medical resource allocation and cost-effectiveness considerations. It is worth noting that this investigation was specifically designed to evaluate the standalone diagnostic performance of chest CT in the discrimination of benign and malignant pulmonary nodules, without the intent to perform a direct head-to-head comparison between chest CT and other imaging modalities, including PET/CT. Consequently, the study framework was centered on chest CT imaging features as the primary investigative parameter, and PET/CT was not included in the comparative analytical design. Collectively, this study confirmed that chest CT remains the first-line, most universally accessible imaging tool for pulmonary nodule screening and initial evaluation in both primary care and routine clinical settings, whereas PET/CT is more appropriately indicated as a second-line modality for risk stratification and patient triage in settings with adequate medical resources. We also completely acknowledge the inherent diagnostic limitations of chest CT as a solitary imaging modality. For indeterminate pulmonary nodules characterized by overlapping benign and malignant imaging features that preclude definitive radiological characterization, morphological evaluation based solely on chest CT is associated with inherent diagnostic constraints. To address this clinical dilemma, this study further integrated a comprehensive multimodal diagnostic algorithm in the “Discussion” section, incorporating validated clinical risk prediction models (Mayo, Brock, and Herder models), liquid biopsy-based molecular detection, and navigational bronchoscopy for minimally invasive tissue diagnosis. All of the findings highlighted that this multi-component, integrated diagnostic strategy can significantly enhance diagnostic confidence for diagnostically challenging pulmonary nodules and further optimize the evidence-based clinical framework for the holistic diagnosis and longitudinal management of pulmonary nodules.

In recent years, artificial intelligence-assisted computed tomography image analysis and radiomic models have been considered innovative technical pillars that facilitate the precise diagnosis of pulmonary nodules. Deep learning algorithms have demonstrated considerable promise in the quantitative characterization of nodule morphological traits and automated malignant risk stratification. However, these predictive models are still hindered by notable practical limitations, including limited external generalizability, incomplete clinical regulatory validation, and poor interoperability with standard radiological diagnostic workflows. Therefore, they are still in the developmental phase of clinical optimization and translational implementation. The robust diagnostic performance data for chest CT generated in this study may also provide a valuable reference baseline for future comparative investigations that involve artificial intelligence models, radiomic signatures, and multimodal combined diagnostic algorithms.

This study has several limitations. First, significant heterogeneity was observed across the included studies, which was primarily attributed to variations in scanning parameters, slice thickness, and post-processing algorithms. Such heterogeneity may have led to an overestimation of the diagnostic performance of chest CT. Second, a substantial proportion of the enrolled studies were retrospective, single-center investigations with relatively small sample sizes, which may introduce selection bias and limit the robustness and generalizability of the pooled estimates. Third, the inability to perform a feature-level meta-analysis represents a significant constraint. This study was unable to perform a feature-level meta-analysis (e.g., spiculation, calcification patterns, ground-glass vs. solid components) due to inconsistent reporting across the included studies. Future primary studies should standardize the documentation of morphological features to enable more granular meta-analytic evaluation. Although this meta-analysis focused on overall diagnostic performance at the patient level, it is important to contextualize our findings with established imaging characteristics. Spiculation, lobulation, and part-solid morphology are well-documented malignancy indicators. The pooled estimates likely reflect the aggregate diagnostic yield of these features to some extent, although individual feature analysis was precluded due to insufficient reporting in primary studies. Fourth, the majority of the included studies were retrospective in design, and several domains in the QUADAS-2 assessment were rated as unclear or high risk of bias, particularly regarding patient selection and blinding of index test interpretation. This may have led to an overestimation of diagnostic accuracy and should be considered when interpreting the pooled estimates. Fifth, CT-based decision-making remains uncertain (e.g., subsolid nodules with indeterminate features and small nodules in high-risk patients). In such circumstances, joint decision-making that incorporates patient preferences is essential. Sixth, owing to substantial heterogeneity in study design, methodological approaches, and CT acquisition protocols across the included literature, valid diagnostic thresholds could not be extracted from a considerable number of investigations. Moreover, there are significant differences among the obtained threshold values, reflecting variations across different studies. Consequently, further subgroup analyses based on diagnostic thresholds were not feasible in this meta-analysis. Seventh, although Chinese and English literature were systematically searched and included, the potential for residual publication bias cannot be fully excluded. Furthermore, we did not conduct head-to-head comparative analyses of advanced imaging modalities, including dual-energy CT, spectral CT, perfusion CT, PET/CT, and radiomic-based CT, all of which are increasingly utilized in the characterization of pulmonary nodules. To overcome these limitations, future large-sample, multicenter, prospective studies with standardized scanning protocols and unified diagnostic thresholds across institutions should be conducted. The integration of artificial intelligence algorithms and quantitative imaging analysis is expected to further enhance the diagnostic accuracy, objectivity, and reproducibility of CT in differentiating benign from malignant pulmonary nodules, which, in turn, may provide more robust and reliable evidence to support clinical decision-making in the future.

## Data Availability

The original contributions presented in the study are included in the article/Supplementary material, further inquiries can be directed to the corresponding author.

## References

[1] Walter K. Pulmonary nodules. JAMA. (2021) 326:1544. doi: 10.1001/jama.2021.12319, 34665202

[2] Yang X He J Wang J Li W Liu C Gao D . CT-based radiomics signature for differentiating solitary granulomatous nodules from solid lung adenocarcinoma. Lung Cancer. (2018) 125:109–14. doi: 10.1016/j.lungcan.2018.09.013, 30429007

[3] Bray F Laversanne M Sung H Ferlay J Siegel RL Soerjomataram I . Global cancer statistics 2022: GLOBOCAN estimates of incidence and mortality worldwide for 36 cancers in 185 countries. CA Cancer J Clin. (2024) 74:229–63. doi: 10.3322/caac.21834, 38572751

[4] Awaya H Matsumoto T Honjo K Miura G Emoto T Matsunaga N. A preliminary study of discrimination among the components of small pulmonary nodules by MR imaging: correlation between MR images and histologic appearance. Radiat Med. (2000) 18:29–38. 10852653

[5] Liu H Chen R Tong C Liang XW. MRI versus CT for the detection of pulmonary nodules: a meta-analysis. Medicine (Baltimore). (2021) 100:e27270. doi: 10.1097/MD.0000000000027270, 34678861 PMC8542155

[6] Martini K Eberhard M Frauenfelder T. Lungenrundherde – Ein Überblick [pulmonary nodules - an overview]. Ther Umsch. (2020) 77:75–80. doi: 10.1024/0040-5930/a001156, 32633224

[7] Huang QX Huang XW. QUADAS-2 tool for quality assessment in diagnostic meta-analysis. Ann Palliat Med. (2022) 11:1844–5. doi: 10.21037/apm-22-204, 35400153

[8] Swensen SJ Morin RL Schueler BA Brown LR Cortese DA Pairolero PC . Solitary pulmonary nodule: CT evaluation of enhancement with iodinated contrast material--a preliminary report. Radiology. (1992) 182:343–7. doi: 10.1148/radiology.182.2.1732947, 1732947

[9] Yamashita K Matsunobe S Tsuda T Nemoto T Matsumoto K Miki H . Solitary pulmonary nodule: preliminary study of evaluation with incremental dynamic CT. Radiology. (1995) 194:399–405. doi: 10.1148/radiology.194.2.7824717, 7824717

[10] Swensen SJ Brown LR Colby TV Weaver AL. Pulmonary nodules: CT evaluation of enhancement with iodinated contrast material. Radiology. (1995) 194:393–8. doi: 10.1148/radiology.194.2.7824716, 7824716

[11] Swensen SJ Brown LR Colby TV Weaver AL Midthun DE. Lung nodule enhancement at CT: prospective findings. Radiology. (1996) 201:447–55. doi: 10.1148/radiology.201.2.8888239, 8888239

[12] Swensen SJ Viggiano RW Midthun DE Müller NL Sherrick A Yamashita K . Lung nodule enhancement at CT: multicenter study. Radiology. (2000) 214:73–80. doi: 10.1148/radiology.214.1.r00ja1473, 10644104

[13] Potente G Iacari V Caimi M. The challenge of solitary pulmonary nodules: HRCT evaluation. Comput Med Imaging Graph. (1997) 21:39–46. doi: 10.1016/S0895-6111(96)00071-7, 9118069

[14] Zhang M Kono M. Solitary pulmonary nodules: evaluation of blood flow patterns with dynamic CT. Radiology. (1997) 205:471–8. doi: 10.1148/radiology.205.2.9356631, 9356631

[15] Yi CA Lee KS Kim EA Han J Kim H Kwon OJ . Solitary pulmonary nodules: dynamic enhanced multi-detector row CT study and comparison with vascular endothelial growth factor and microvessel density. Radiology. (2004) 233:191–9. doi: 10.1148/radiol.2331031535, 15304661

[16] Kim JH Kim HJ Lee KH Kim KH Lee HL. Solitary pulmonary nodules: a comparative study evaluated with contrast-enhanced dynamic MR imaging and CT. J Comput Assist Tomogr. (2004) 28:766–75. doi: 10.1097/00004728-200411000-00007, 15538149

[17] Jeong YJ Lee KS Jeong SY Chung MJ Shim SS Kim H . Solitary pulmonary nodule: characterization with combined wash-in and washout features at dynamic multi-detector row CT. Radiology. (2005) 237:675–83. doi: 10.1148/radiol.2372041549, 16244276

[18] Orlacchio A Schillaci O Antonelli L D’ Urso S Sergiacomi G Nicolì G . Nodulo polmonare solitario: caratterizzazione morfologico-metabolica mediante imaging integrato TCms/FDG-PET. Radiol Med. (2007) 112:157–73. doi: 10.1007/s11547-007-0132-x 17361379

[19] Lee KS Yi CA Jeong SY Jeong YJ Kim S Chung MJ . Solid or partly solid solitary pulmonary nodules: their characterization using contrast wash-in and morphologic features at helical CT. Chest. (2007) 131:1516–25. doi: 10.1378/chest.06-2526 17494800

[20] Ohno Y Koyama H Takenaka D Nogami M Maniwa Y Nishimura Y . Dynamic MRI, dynamic multidetector-row computed tomography (MDCT), and coregistered 2-[fluorine-18]-fluoro-2-deoxy-D-glucose-positron emission tomography (FDG-PET)/CT: comparative study of capability for management of pulmonary nodules. J Magn Reson Imaging. (2008) 27:1284–95. doi: 10.1002/jmri.21348, 18504748

[21] Choi EJ Jin GY Han YM Lee YS Kweon KS. Solitary pulmonary nodule on helical dynamic CT scans: analysis of the enhancement patterns using a computer-aided diagnosis (CAD) system. Korean J Radiol. (2008) 9:401–8. doi: 10.3348/kjr.2008.9.5.401, 18838848 PMC2627214

[22] Bayraktaroglu S Savaş R Basoglu ÖK Cakan A Mogulkoc N Cagrc U . Dynamic computed tomography in solitary pulmonary nodules. J Comput Assist Tomogr. (2008) 32:222–7. doi: 10.1097/RCT.0b013e318136e29d, 18379306

[23] Bai RJ Cheng XG Qu H Shen BZ Han MJ Wu ZH. Solitary pulmonary nodules: comparison of multi-slice computed tomography perfusion study with vascular endothelial growth factor and microvessel density. Chin Med J. (2009) 122:541–7. doi: 10.3760/cma.j.issn.0366-6999.2009.05.011 19323905

[24] Jiang NC Han P Zhou CK Zheng JL Shi HS Xiao J. Dynamic enhancement patterns of solitary pulmonary nodules at multi-detector row CT and correlation with vascular endothelial growth factor and microvessel density. Ai Zheng. (2009) 28:164–9. Available online at: https://kns.cnki.net/kcms2/article/abstract?v=lj-1FT9NYjAkyFC-LL6sQWqLCdxAR30QlnSzheBIIN3Bl0ZPGR1fKYnAWK_lPMFQeD1BqyBM5_ce6QpoOtlnStqRuMpuPW-HPDgB2wy4RWOL5-9kkWwuNfk8Bt5aQLZiXqGHx1GhnIuZpzmIOwwTjth6iMasgrE-V-bhMxWbwlK6K-Oqmt74sQ==&uniplatform=NZKPT&language=CHS 19550130

[25] Dabrowska M Zukowska M Krenke R Domagała-Kulawik J Maskey-Warzechowska M Bogdan J . Simplified method of dynamic contrast-enhanced computed tomography in the evaluation of indeterminate pulmonary nodules. Respiration. (2010) 79:91–6. doi: 10.1159/000213760, 19372641

[26] Li Y Yang ZG Chen TW Yu JQ Sun JY Chen HJ. Firstpass perfusion imaging of solitary pulmonary nodules with 64-detector row CT: comparison of perfusion parameters of malignant and benign lesions. Br J Radiol. (2010) 83:785–90. doi: 10.1259/bjr/58020866, 20647512 PMC3473400

[27] Ohno Y Koyama H Matsumoto K Onishi Y Takenaka D Fujisawa Y . Differentiation of malignant and benign pulmonary nodules with quantitative firstpass 320-detector row perfusion CT versus FDG PET/CT. Radiology. (2011) 258:599–609. doi: 10.1148/radiol.10100245, 21273522

[28] Ohno Y Nishio M Koyama H Fujisawa Y Yoshikawa T Matsumoto S . Comparison of quantitatively analyzed dynamic area-detector CT using various mathematic methods with FDG PET/CT in management of solitary pulmonary nodules. AJR Am J Roentgenol. (2013) 200:W593–602. doi: 10.2214/AJR.12.9197, 23701089

[29] Ohno Y Nishio M Koyama H Seki S Tsubakimoto M Fujisawa Y . Solitary pulmonary nodules: comparison of dynamic first-pass contrast-enhanced perfusion area-detector CT, dynamic first-pass contrast-enhanced MR imaging, and FDG PET/CT. Radiology. (2015) 274:563–75. doi: 10.1148/radiol.14132289, 25203128

[30] Shu SJ Liu BL Jiang HJ. Optimization of the scanning technique and diagnosis of pulmonary nodules with first-pass 64-detector-row perfusion VCT. Clin Imaging. (2013) 37:256–64. doi: 10.1016/j.clinimag.2012.05.004, 23465977

[31] Ribeiro SM Ruiz RL Yoo HH Cataneo DC Cataneo AJ. Proposal to utilize simplified swensen protocol in diagnosis of isolated pulmonary nodule. Acta Radiol. (2013) 54:757–64. doi: 10.1177/0284185113481695, 23550185

[32] Li XY Zhang XL Zhang YZ Chen B Wang JP Shi Z. Dynamic enhanced scanning of the pulmonary nodules with 16-slice spiral CT (in Chinese). J South Med Univ. (2009) 29:133–6. Available online at: https://kns.cnki.net/kcms2/article/abstract?v=lj-1FT9NYjAVqXtQ5cU6jFnyBypPqXCPG0fKLrz51FuQKtK5S7fwBpcV7L-bLXj2qz0LRpj63WvD5Z2NbfT-PWF8f4T2IOBQbhosmXn7X4DTLT7L7-jCHzzAIGnDQMWERIXXKf3pP7Jf0NiIn3KWBSiTPJ4udgWAEZRTrPENKZFkiLE8tFBr3Q==&uniplatform=NZKPT&language=CHS 19218133

[33] Fan L Liu SY Li QC Yu H Xiao XS. Multidetector CT features of pulmonary focal ground-glass opacity: differences between benign and malignant. Br J Radiol. (2012) 85:897–904. doi: 10.1259/bjr/33150223, 22128130 PMC3474071

[34] Chen W Liu J Li W Xiong Z Zhu Z. Value of the likelihood ratio in the CT qualitative diagnosis of solitary pulmonary nodules (in Chinese). Radiol Pract. (2009) 24:727–31. doi: 10.13609/j.cnki.1000-0313.2009.07.034

[35] da Silva EC Silva AC de Paiva AC Nunes RA Gattass M. Diagnosis of solitary lung nodules using the local form of Ripley's K function applied to three-dimensional CT data. Comput Methods Prog Biomed. (2008) 90:230–9. doi: 10.1016/j.cmpb.2008.02.003, 18403042

[36] Ding Y Zhang L Qian X Zhai R. Perfusion imaging with 64-slice spiral CT in differential diagnosis of solitary pulmonary nodule (in Chinese). Chin J Med Imaging Technol. (2007) 25:214–8. Available online at: https://kns.cnki.net/kcms2/article/abstract?v=lj-1FT9NYjCGjZBZ-ot2DejZ_72VoUmxJOIm9WffnqIEuh_syIC4hX84ltObjxUYjWxCTI9gtTIo3f-p0jaoAATgzEcSnZ5Je26NW2vEbY3d0T2k423MW2a7RlDZ5UQlBM8k6WeFDjcAntWHWEdu0DuYPIMoXCx8MAHLte2_POankb0lB-eCyA==&uniplatform=NZKPT&language=CHS

[37] Han X Yang S Zhang H Zhou A Liang P Yang Q. Differential diagnosis of solitary pulmonary nodules by using single location dynamic enhanced CT (in Chinese). J Jilin Univ Med Ed. (2009) 25:929–32. Available online at: https://kns.cnki.net/kcms2/article/abstract?v=lj-1FT9NYjCEOCYIHMbi3XbR4tvIImpV0Ex2Z1GarLSSqpLPNaVHFD1f1m-E65WpMmpk28sNFHGKcY4v9mTd-JO-ScQ0gIfyw6iygKa7blxChHkObRAmgLEqUDBeVmb_Haan3xJUfb9YBMfOsHtlm_5Rst0Dvx2ZfxjY0RyiHDPhVaHhqeeiAg==&uniplatform=NZKPT&language=CHS

[38] Huang Y Wu N Lin DM Li L Wang JW. The diagnostic value of dynamic CT in solitary pulmonary nodules: a prospective study (in Chinese). Zhonghua Zhong Liu Za Zhi. (2005) 27:360–3. 16117900

[39] Iwano S Nakamura T Kamioka Y Ikeda M Ishigaki T. Computeraided differentiation of malignant from benign solitary pulmonary nodules imaged by high-resolution CT. Comput Med Imaging Graph. (2008) 32:416–22. doi: 10.1016/j.compmedimag.2008.04.001, 18501556

[40] Liu L Liu W Chu C Wu J Zhou Y Zhang H . Chest CT imaging on differentiating malignant form benign solitary pulmonary nodules (in Chinese). Opt Precis Eng. (2009) 17:2066–8. Available online at: https://kns.cnki.net/kcms2/article/abstract?v=lj-1FT9NYjA9X-p63Zup_DWWR70oFgUSqYp1p40mc5XN9Fq4juuOpmZR_Lh71U-J7gd2wOcnAKjR0PmEA2lLdnAKEurmMvwQzjacb0H_LYL1CjrgBF9CFFCe-uNwJulgtyYFumdJFMhSCmU3_FB8IHZVNNQDMo-pHuPmuei4MPFxG96TNe3fqQ==&uniplatform=NZKPT&language=CHS

[41] Song X Wang G Zhao Z. Diagnostic value of CT and MRI in solitary pulmonary nodules (in Chinese). Chin Med J Metall Ind. (2006) 23:27–9. doi: 10.13586/j.cnki.yuyx1984.2006.01.002

[42] Sun J Sun C Yang Z Zhou X Zhou Q Che G . Differential diagnosis for benign and malignant solitary pulmonary nodules: comparison of the first pass dynamic and delayed contrast CT (in Chinese). J Clin Radiol. (2008) 28:781–5. doi: 10.13437/j.cnki.jcr.2008.06.048

[43] Wen J Mao L Sun M. Clinical analysis on 74 cases of solitary pulmonary nodules (in Chinese). J Dalian Med Univ. (2007) 29:46–7. Available online at: https://kns.cnki.net/kcms2/article/abstract?v=lj-1FT9NYjCxH08y7Cnm6HcsRwrH7fdqQaYJLiBfsRGD1rQrb1s2N6omFYkfFxLwuvG3UKymqyFs4Is-1x8QuUc0KKDNgrgP45ttQqUULafV2yKKkwmenFghl33qziAQXjy-6VX1RkqNclWH1gQSHD0I11f_pL04MaPZOVk9CNGr3ZtYFVRbrQ==&uniplatform=NZKPT&language=CHS

[44] Ye XD Ye JD Yuan Z Li WT Xiao XS. Dynamic CT of solitary pulmonary nodules: comparison of contrast medium distribution characteristic of malignant and benign lesions. Clin Transl Oncol. (2014) 16:49–56. doi: 10.1007/s12094-013-1039-8, 23606354

[45] Li C Liu B Meng H Lv W Jia H. Efficacy and radiation exposure of ultra-low-dose chest CT at 100 kVp with tin filtration in CT-guided percutaneous Core needle biopsy for small pulmonary lesions using a third-generation dual-source CT scanner. J Vasc Interv Radiol. (2019) 30:95–102. doi: 10.1016/j.jvir.2018.06.013, 30149997

[46] Meng XX Kuai XP Dong WH Jia NY Liu SY Xiao XS. Comparison of lung lesion biopsies between low-dose CT-guided and conventional CT-guided techniques. Acta Radiol. (2013) 54:909–15. doi: 10.1177/0284185113485937, 23817682

[47] Fu YF Li GC Xu QS Shi YB Wang C Wang T. Computed tomography-guided lung biopsy: a randomized controlled trial of low-dose versus standard-dose protocol. Eur Radiol. (2020) 30:1584–92. doi: 10.1007/s00330-019-06464-6, 31776740

[48] Li EL Ma AL Wang T Fu YF Liu HY Li GC. Low-dose versus standard-dose computed tomography-guided biopsy for pulmonary nodules: a randomized controlled trial. J Cardiothorac Surg. (2023) 18:86. doi: 10.1186/s13019-023-02183-8, 36927419 PMC10018993

[49] Wu R Lu H Chang W. Diagnosis value of enhanced helical CT scanning on solitary pulmonary nodules (in Chinese). Prog Mod Biomed. (2007) 7:259–60. Available online at: https://kns.cnki.net/kcms2/article/abstract?v=lj-1FT9NYjCjjmkDWLNt-BX4gh8JFOu5uwCuYd4W95gtpZEe1IkZ8bQbImJ1gEXsIU8gprP99zxgkEMXIowbHzLf10PrgjQBc2uwQjCaAmtJlen4mdMBlxCWqPF90vumZCrxZdxohTdH2t4l8kqglhmXOkoLX0oP8nZEyrhhZp7K6knAx5Oeg==&uniplatform=NZKPT&language=CHS

[50] Yang G Yao S Huang K Ning G Liu S. The diagnostic value in solitary pulmonary nodules with thin slice spiral CT and MPVR reconstruction imaging (in Chinese). J Med Imaging. (2007) 17:790–2. Available online at: https://kns.cnki.net/kcms2/article/abstract?v=lj-1FT9NYjCwt-9u9MSL1QGfwqrWwfxkMlm_LQIwjHSSP29XrCWoXF82big9X7m4XEzueiDRmzWXZBvMvQUbuEAXSh73i7oAehwlAs7tre3dSMd1R0a5Y-ZG8pJ3lwA5EP17eVcS9JeJXDtCDn4aI4mMjGSyO8jkAOennibC0EAq5wTrVKdqvg==&uniplatform=NZKPT&language=CHS

[51] Zhang M Zhou H Zou Y. Quantitative investigation of solitary pulmonary nodules with dynamic contrast enhanced functional CT (in Chinese). Chin J Radiol. (2004) 38:263–7. Available online at: https://kns.cnki.net/kcms2/article/abstract?v=lj-1FT9NYjB2QRgj3uoMOOURgceTUiXAcgJHXm6erxRzORBKkeQszDU-m5RXySLAb0s3N6EWSgyKA9q1IJVxWGBEnaSuSE9oT6MZtu9YkBmZWlmvvNH2POakSjDwRvXqOf-ZeSpjBFFJ3KBDmZ51hidPgoFl7XM-GdWyMkQ1otyAD-pt_EZkWA==&uniplatform=NZKPT&language=CHS

[52] Zou Y Wen Z Jiang L Alifu Z Zhao Y Nuerlan T. Diagnosis and differentiation of solitary pulmonary nodules with multi-slice spiral CT (in Chinese). J Xinjiang Med Univ. (2009) 32:1486–9. Available online at: https://kns.cnki.net/kcms2/article/abstract?v=lj-1FT9NYjBRdICfv79dMaGO3Ns5gtcdqZVgnQhjjEo228YHNE37_j_9H_Ttkl-XJB_KFc0iLdQ6U570E3phj5qM7JuNT8iKdcWMgKSDZbI5BjT49cMK222D4FJZvNRCnlf7CxfeFHbLoN2cz6Dl1S_YU_F49dVeH37iX1ihRkJix9W0BVvdag==&uniplatform=NZKPT&language=CHS

[53] Cui H. Application analysis of MSCT low-dose scanning in the diagnosis of pulmonary nodules. Mod Diagn Treat. (2021) 32:619–21. Available online at: https://kns.cnki.net/kcms2/article/abstract?v=lj-1FT9NYjDif3Ez67CAKeHO7PUPMfcs-_n7dAtI2mZn_bRdx-vuCWCqxH4FC0u4OsLWFLZiBJJBWBjuUUBUrGZGwLY3phN_1e1ZJ2MrJY1siHElnRGEUgdnUNAD4yKFlQO2Op8srhJbZROhVVTDuuryXp2YrOSp0Jb-_dHeABd0-nxsvA4Unw==&uniplatform=NZKPT&language=CHS

[54] Zhang CY Yu HL Li X Sun YY. Diagnostic value of computed tomography scanning in differentiating malignant from benign solitary pulmonary nodules: a meta-analysis. Tumour Biol. (2014) 35:8551–8. doi: 10.1007/s13277-014-2113-8, 24859887

[55] Wu Q Zhong L Xie X. The value of four imaging modalities to distinguish malignant from benign solitary pulmonary nodules: a study based on 73 cohorts incorporating 7956 individuals. Clin Transl Oncol. (2021) 23:296–310. doi: 10.1007/s12094-020-02418-3, 32548796

[56] Qureshi NR Shah A Eaton RJ Miles K Gilbert FJ Sputnik investigators. Dynamic contrast enhanced CT in nodule characterization: how we review and report. Cancer Imaging. (2016) 16:16. doi: 10.1186/s40644-016-0074-4 27430260 PMC4948091

