# Supplementary figures and images for: The value of chest CT in the differential diagnosis of benign and malignant pulmonary nodules: a meta-analysis
Source: Front Med (Lausanne). 2026 May 28;13:1854231. doi: 10.3389/fmed.2026.1854231 (PMC13270085; doi:10.3389/fmed.2026.1854231)

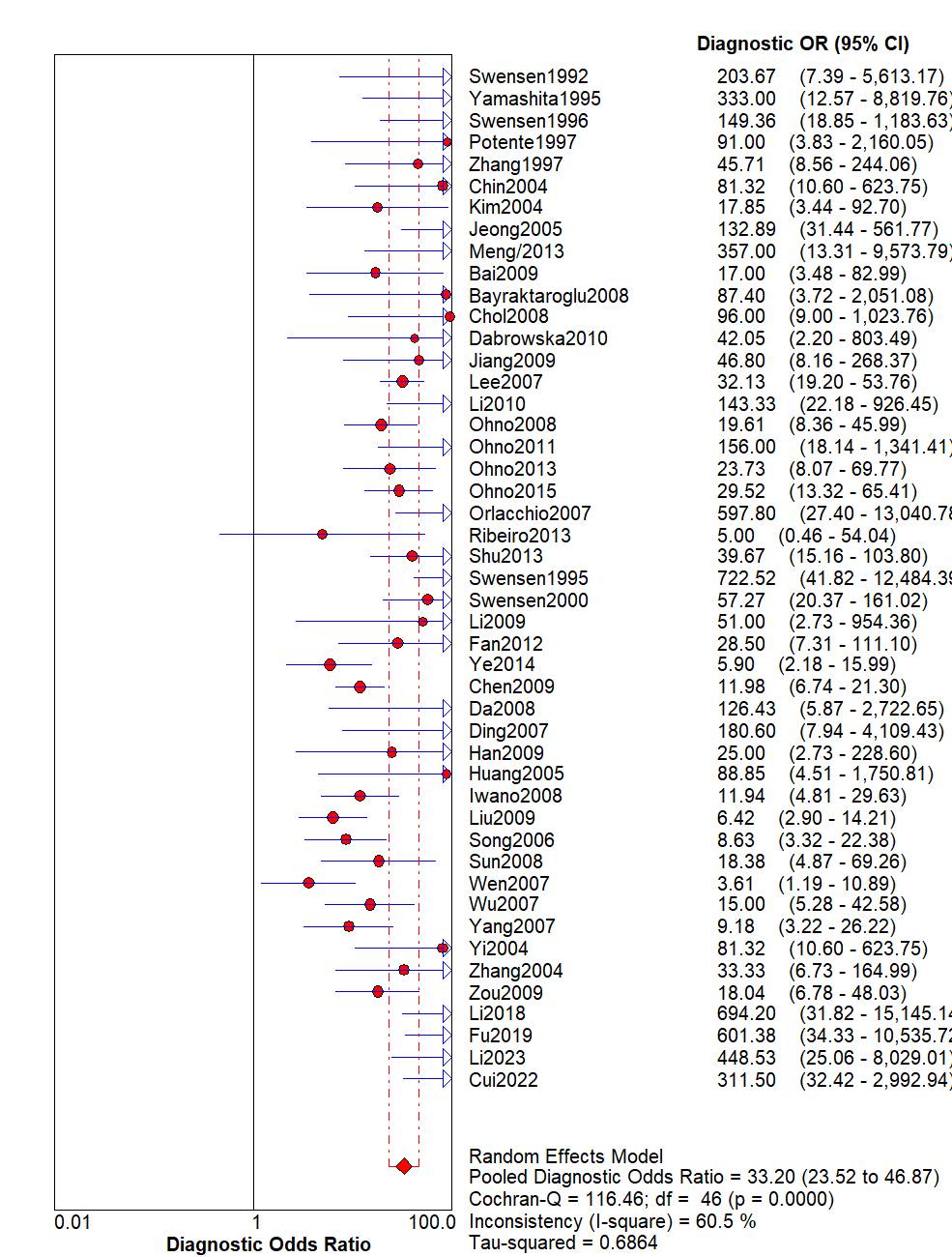

Supplement: Supplementary file 1 [file Image_1.jpeg]

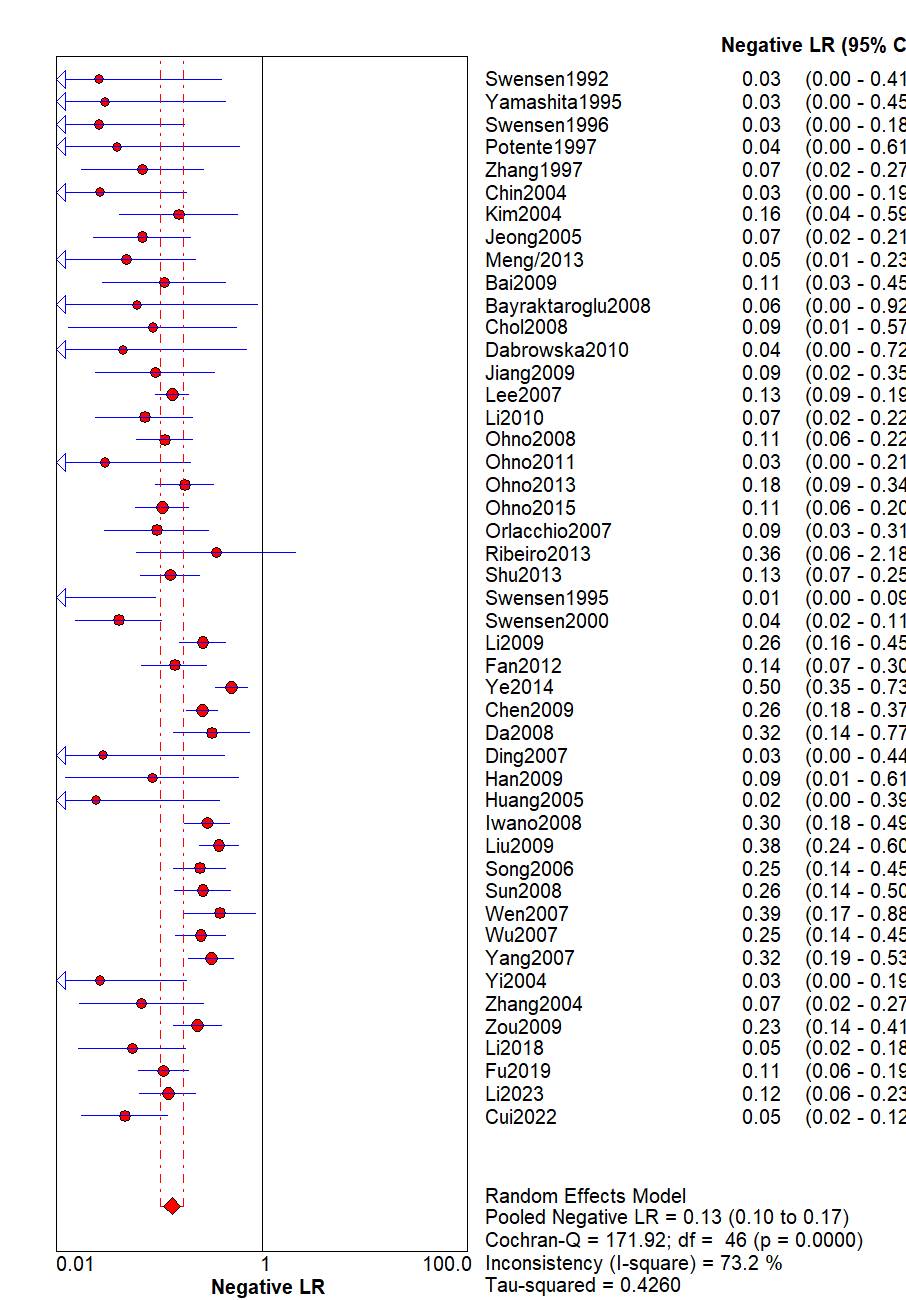

Supplement: Supplementary file 2 [file Image_2.jpeg]

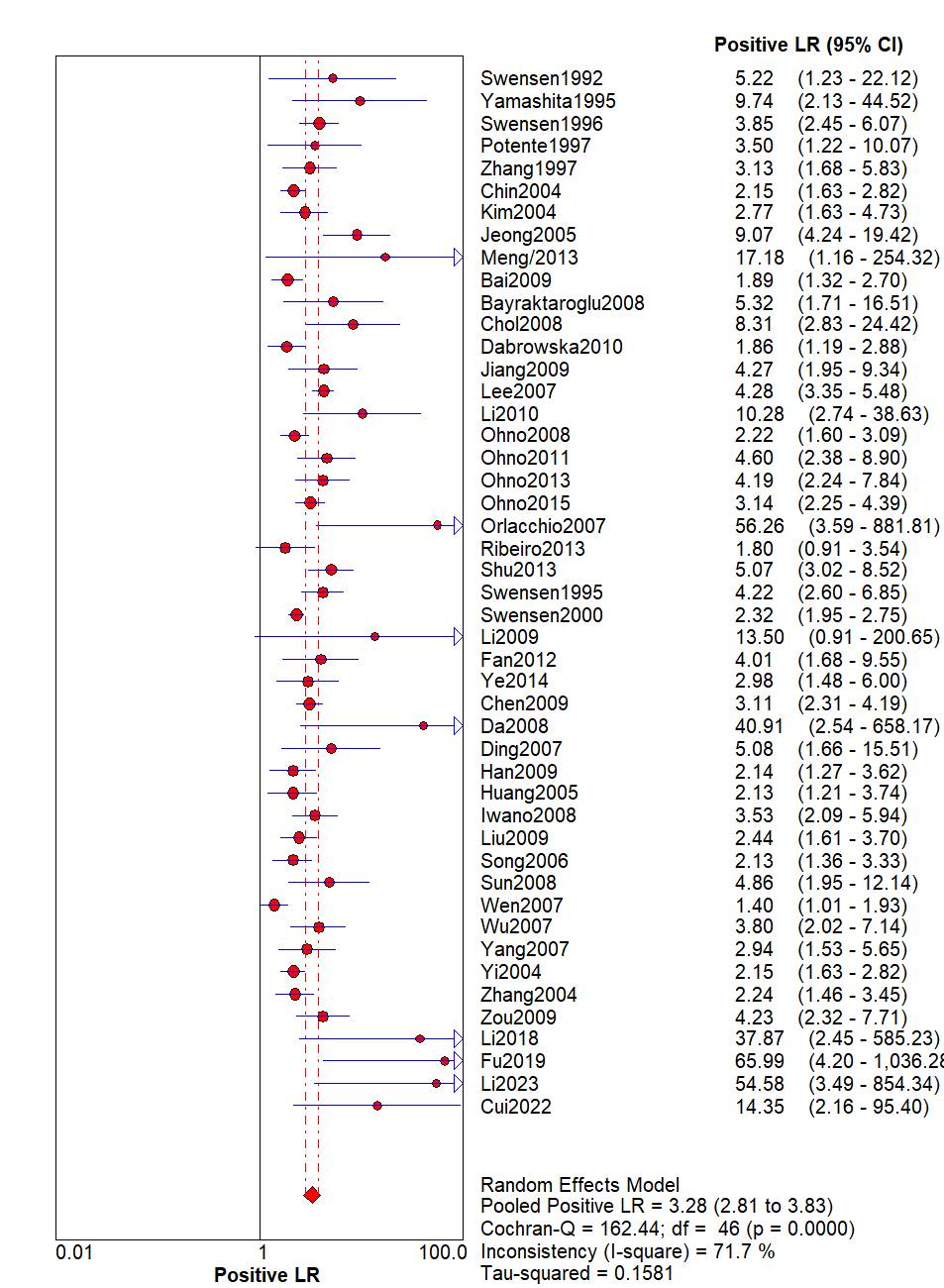

Supplement: Supplementary file 3 [file Image_3.jpeg]
